# Hand Hygiene Intervention Strategies to Reduce Diarrhoea and Respiratory Infections among Schoolchildren in Developing Countries: A Systematic Review

**DOI:** 10.3390/ijerph14040371

**Published:** 2017-04-01

**Authors:** Balwani Chingatichifwe Mbakaya, Paul H. Lee, Regina L. T. Lee

**Affiliations:** 1 School of Nursing, The Hong Kong Polytechnic University, Hung Hom, Kowloon, Hong Kong, SAR, China; balwani-mbakaya.chingatichifwe@connect.polyu.hk; 2 World Health Organization Collaborating Centre for Community Health Services, School of Nursing, The Hong Kong Polytechnic University, Hung Hom, Hong Kong, SAR, China; paul.h.lee@polyu.edu.hk

**Keywords:** multi-level intervention, hand washing, strategies, schoolchildren, diarrhoea, respiratory infections, developing countries

## Abstract

Effective and appropriate hand-washing practice for schoolchildren is important in preventing infectious diseases such as diarrhoea, which is the second most common cause of death among school-age children in sub-Saharan Africa. The objective of the review was to identify hand hygiene intervention strategies to reduce infectious diseases such as diarrhoea and respiratory tract infections among schoolchildren aged 6–12 years in developing countries. Published research articles were searched from databases covering a period from as far back as the creation of the databases to November 2015. Eight randomized controlled trials (RCT/CRCT) from developing countries met the inclusion criteria. The Jadad Scale for appraising RCT/CRCT studies revealed methodological challenges in most studies, such that 75% (6/8) were rated as low-quality articles. The review found that hand hygiene can reduce the incidence of diarrhoea and respiratory conditions. Three hand hygiene intervention strategies utilized were training, funding and policy, with training and funding implemented more commonly than policy. These strategies were not only used in isolation but also in combination, and they qualified as multi-level interventions. Factors that influenced hand washing were contextual, psychosocial and technological. Findings can inform school health workers in categorizing and prioritizing activities into viable strategies when implementing multi-level hand-washing interventions. This review also adds to the existing evidence that multi-level hand-washing interventions can reduce the incidence of diarrhoea, respiratory infections, and school absenteeism. Further evidence-based studies are needed with improved methodological rigour in developing countries, to inform policy in this area.

## 1. Introduction

Effective and appropriate hand hygiene practice for schoolchildren is important in preventing infectious diseases such as diarrhoea, which is the second most common cause of death among school-age children in sub-Saharan Africa [1]. Because lifestyle and behavioural choices are made in childhood, it is important that health education about hand hygiene be introduced very early to influence healthy behaviours [2]. This is possible to achieve in children because their poor hygiene habits are less established, unlike adults, whose habits are firmly grounded and difficult or unlikely to change [3]. Well-practiced and consistent hand-washing technique/skill can produce significant benefits in reducing incidence of gastro-intestinal and respiratory infections [4]. Proper hand washing with soap can reduce the risk of diarrhoea by 42%–48% [4]. In turn, this can lead to reductions in morbidity and mortality rates, as well as in school absenteeism among children [5]. Consequently, this may lead to an improvement in their school performance, which may in the end have positive implications for development in their countries [6]. Studies have revealed that students who are absent frequently or for long periods are likely to have difficulty mastering the material presented in class, making absenteeism an important education issue [6]. Therefore, hand washing has the simultaneous benefit of improving both education and health [7]. Unfortunately, evidence of scientifically sound studies such as randomized controlled trials is inadequate in developing countries [8], even though the incidence of infectious diseases among schoolchildren in these countries is very high [3].

A recent review by Willmott and colleagues [8] summarizes evidence on the effectiveness of hand hygiene interventions in reducing infectious illness and/or absence in educational settings for children aged three to eleven years and/or staff working with them, and obtains a quantified estimate of the effect [9]. However, the majority (11/18) of the referenced studies are from developed countries, resulting in the review [9] having less focus on developing countries, where the morbidity and mortality from infectious disease in children is the highest and contributes to most child deaths [3]. In addition, the recent systematic review [9] did not distinguish between hand washing with soap and using hand sanitizer, even though the two may have different effectiveness and resource implications. Furthermore, its focus was not only on schoolchildren but combined with staff working with them. The present review extends this work by updating the findings, with a greater focus on hand washing among schoolchildren in developing countries. It further identifies the multi-level interventions and strategies used to reduce infectious diseases, such as diarrhoea and respiratory tract infections among schoolchildren, and distinguishes between the effectiveness of hand washing with soap and with hand sanitizer. Finally, factors that influence hand washing among schoolchildren in developing countries are highlighted. The main objective of the systematic review was to identify hand hygiene intervention strategies to reduce infectious diseases among schoolchildren aged six to twelve years in developing countries. This review was conducted to answer the following questions: (1) What are the hand hygiene intervention strategies used to reduce diarrhoea and respiratory infections among schoolchildren in developing countries? (2) What are the factors that influence hand hygiene practices among schoolchildren in developing countries? (3) Are multi-level interventions used/applied in hand hygiene interventions implemented among schoolchildren in developing countries?

In this review, the concepts of multi-level interventions and strategies were used to describe and categorize the activities/interventions used in the studies under review. For an intervention to be considered multi-level, it has to address at least three different sources of influence [10,11]. While a multi-level intervention is less robust in health care, it is believed that multiple levels influence interdependent interaction, thereby producing desirable outcomes and helping to improve the adoption of an intervention [10]. Multi-level interventions are currently recommended for use in health care because client/patient outcomes are a primary measure by which we assess healthcare delivery quality, and these outcomes are influenced by numerous other factors in the multi-level context of care [11].

## 2. Materials and Methods

### 2.1. Inclusion Criteria

The article inclusion criteria were as follows: (1) studies whose participants were schoolchildren aged six to twelve years; (2) studies conducted in developing countries [12]; (3) RCT/CRCT studies; and (4) studies whose outcomes were reduction in incidence of diarrhoea, and/or respiratory illnesses, and/or reduction in school absenteeism, and/or improved cleanliness of hands due to the impacts of hand hygiene, and/or reduction in the number of microorganisms on the hands, which is a proxy measure of hand-washing practice assessed through collection and microbial analysis of hand rinse samples. Papers written in languages other than English were excluded. Special attention was given to papers that did not meet the inclusion criteria because of their wider age range (less than six years and more than twelve years). If the schoolchildren’s age was within the included range, the article was reviewed. However, the data was interpreted with caution. An attempt was made to extract the data on older, school-aged children from these studies.

### 2.2. Search Strategy

The following five database sources were used to gather the required information: Pub Med, CINAHL, Web of Science, Psych Info via Pro-Quest, and Medline via EBSCOhost. Efforts were made to identify both published and unpublished interventional studies by manually checking the reference lists of articles that met the inclusion criteria. Efforts were also made to manually identify articles from the reference list of the latest and most current published systematic review [9]. The period covered research from as far back as the creation of the databases to November 2015. The key search words used were: Effect*; Hand hygiene OR Hand wash* OR Hand disinfection; Intervention OR Strategy OR Technique; AND Schoolchildren. Finally, studies that were conducted in developing countries were identified [12].

### 2.3. Data Extraction Process and Data Items

**Figure 1:**
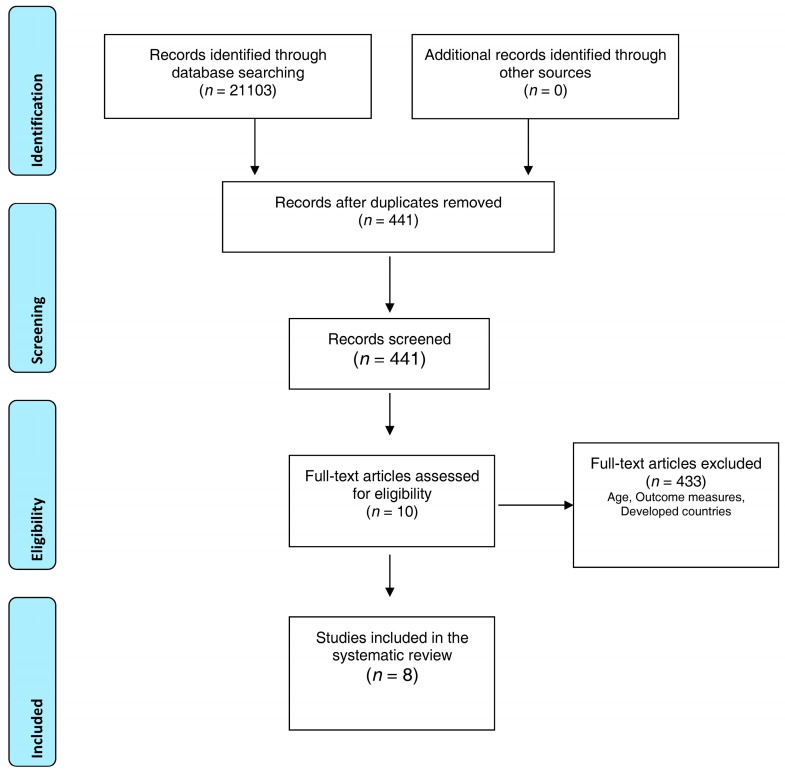
PRISMA flow diagram.

The process of data extraction started with an internet search of relevant articles using search terms while following the PRISMA guidelines (see Figure 1 below). A standardized table was used to guide data extraction from the included papers. All relevant information extracted from each study was summarized and documented. The details included author, year, participants, age, setting, country, intervention, control, cluster and number of clusters (see Table 1 below).

### 2.4. Quality Appraisal

The Jadad scale for reporting RCTs [21] was used to critically appraise the eight studies included in the review. This is a tool that has been validated for use in the critical appraisal of RCTs, and it is favoured for three main reasons. First, it is easy to use compared to other scales/tools. Secondly, it contains many of the important elements that have empirically been shown to correlate with bias. Finally, it has shown reliability and external validity [21]. The Jadad scale has three main components that it assesses, with a total score of five. It assesses randomization, where two marks are awarded; blinding, where another two marks are awarded; and finally it makes sure that an account or the fate of all participants in the trial is known, awarding one more mark to make a total of five. Any score less than three is regarded as low, while a score of three or above is regarded as high [21].

### 2.5. Synthesis of Results

The review identified multi-level interventions and strategies used to reduce diarrhoea and respiratory infections among schoolchildren aged six to twelve in developing countries, as well as distinguishing between the effectiveness of hand washing with soap and with hand sanitizer. For an intervention to be considered multi-level, it has to address at least three different sources of influence [10,11]. For example, apart from the individual client, it has to address at least two levels of contextual influence, such as organization and providers [10,11]. This review further synthesized and categorized hand hygiene interventions and activities used in the articles under review into three strategies, namely training/education, policy, and funding. The authors grouped the interventions into categories to more effectively describe the results. The grouping was based on the common themes of the activities or interventions used in the studies. Factors that influence hand washing among schoolchildren were also identified and described in subsets, as categorized by contextual, psychosocial and technological factors [22,23]. According to Dreibelbis, contextual factors involve determinants related to the individual, setting, and/or environment that can influence behaviour change and the adoption of new technologies; psychosocial factors comprise the behavioural, social, or psychological determinants that influence behavioural outcomes and technology adoption; and technological factors comprise attributes of a technology, product, or device that influence its adoption and sustained use [22,23]. Dreibelbis further stated that these factors influence hand washing at five different aggregate levels: habitual, individual, interpersonal, community, and societal [22]. According to him, the societal/structural level refers to the organizational, institutional, or cultural factors that influence behaviours, such as laws, policies, climate, geography, and distribution of products; the community level includes the physical and social environment in which individuals are nested, as well as the formal and informal institutions that shape individual experiences; the interpersonal/household level represents interactions between individuals and the people they associate with, such as norms, aspirations, shame, sharing access to a product, and behavioural modelling; the individual level includes sociodemographic factors such as age and gender, individual cognitive factors, and attitudes toward the product, hardware, or behaviour; and the habitual level reflects the fact that the opportunity and necessity for hand-washing behaviours are repeated over the course of the day, and the multiple processes or events that can result in the specific behavioural outcomes [22,23]. The narrative synthesis was performed based on content analysis of the included articles. The papers were synthesized and rated, and the results entered a table. 

## 3. Results

### 3.1. Search Outcomes

Initially, a total of 21,103 articles were retrieved from all the databases, based on the key search words. However, only nine unique studies qualified according to the set inclusion criteria. Finally, eight studies were included in the systematic review because one article [19] was duplicated in two databases (Medline and Pub Med). See Table 2 below.

Many papers (20,662) were excluded based on the title and abstract alone, because of non-relevance or duplication. The full texts of the remaining 441 articles were retrieved for more detailed evaluation, and 433 articles were excluded because of age or different outcomes, or because they were done in developed countries (see Figure 1 above). The database search was done by two reviewers (RLTL and BMC) separately, and identified articles that potentially met the inclusion criteria and were independently screened for eligibility. Lack of consensus over the eligibility of an article by the two authors was resolved through discussion with the third reviewer (PHL).

### 3.2. Quality of the Studies

A summary of the quality assessment scores of the included studies using the Jadad Scale for reporting RCTs is presented in Table 3 below. The assessment was performed by two reviewers independently using the Jadad Scale [21]. Any disagreement between the reviewers about the criteria and/or level of bias was discussed until a mutual decision was reached, or was decided with the arbitration of a third reviewer. After subjecting all eight included studies to the Jadad Scale, 75% (6/8) were found to be of low quality and only 25% (2/8) were of high quality. The highest score was four out of five [14,15], and the lowest was one out of five [18]. 

All eight studies stated that they had randomized the allocation of subjects to the intervention and control groups. However, four studies [17,18] did not describe the method used to generate the sequence of randomization, thereby attracting a score of one instead of two per the Jadad Scale.

Blinding was found to be the main challenge in almost all the eight studies included in the review. Six studies blinded neither the participants nor the field workers. However, blinding was partially done in the other two studies [14,15]. In these two studies, blinding among the intervention groups was only partially performed, with neither the participants nor the field workers aware of which group was given antibacterial soap and which received plain soap.

The last scoring item on the Jadad Scale requires that research studies ensure that an account or the fate of all participants in the trial is known, to determine whether they have withdrawn or dropped out, and for what reasons [21]. Five of the studies included in this review managed to score the full one mark. However, three studies [18,19,20] failed to account for the loss of participants in the study, and got a zero score. 

### 3.3. Study Characteristics

All eight studies included in the review were RCTs and CRCTs carried out between 2004 and 2013. The majority (5/8) of the studies were current, as they were conducted in 2012 and 2013. All the studies were carried out in developing countries, as this was one of the inclusion criteria. Four were carried out in Kenya, Africa, two in Pakistan, one in Egypt, Africa and one in Uganda, Africa. In these developing countries, six studies were carried out in elementary school settings and two in squatter settlements. These latter two studies had to be included because their participants were school-age children (<15 years old). In addition, at least one of their outcomes was reduced incidence of diarrhoea in school-age children, which was one of the inclusion criteria for review.

### 3.4. Study Participants

The focus of this review was schoolchildren aged six to twelve years. This is because, under normal circumstances, children start elementary school at the age of six, and at age twelve, they might have just graduated from junior elementary school. However, we took into consideration the fact that in developing countries with resource constraints, some children start primary school late. As such, they may still be in primary school after age twelve, or may repeat classes because of infectious diseases, opportunistic infections due to HIV and AIDS, and other socio-economic challenges. Therefore, if the study participants were schoolchildren, the article was included in the review. All eight studies under review had participants within the elementary school age range, with the oldest being sixteen. Studies whose participants included only children under five were omitted, since these subjects were not of school age.

### 3.5. Study Intervention, Controls and Setting

Five studies used three arms, with two intervention groups and one control group, while three studies used two arms, with one intervention and one control group. Out of the eight interventions, only one [17] compared hand washing with soap to washing with an alcohol-based hand sanitizer and to a control group. Two studies [14,15] compared hand washing with antibacterial soap to washing with plain soap and to a control group. The control group in most studies used either standard practice or no treatment at all. However, two studies [14,19] were not clear on whether the control group was given a placebo, the standard treatment, or no intervention at all. Six studies [13,16,17,18,19,20] used school settings, and two [15,19] used neighbourhood and household settings. 

### 3.6. Study Key Findings

The longest period of follow-up of participants in the reviewed studies was 2 years [16], while the shortest was 12 weeks [19]. As detailed in their methodologies, follow-ups were completed in all the studies under review. Homogeneity was missing from most studies. For example, in one of the studies [18], the two strata were different. The Kisumu/Nyando geographic stratum was generally less rural than that of Rachuonyo, which would end up having implications for the results of the study. On the other hand, there would have been a possibility of contamination (sharing of information) among participants in one of the studies [14], because both control and intervention were living in the same neighbourhood and separated by only a street or market. This in turn would affect the findings of the study due to the Hawthorne effect, because the attention given to the treatment group plus the participants’ interaction may have caused a change in behaviour. Different sample sizes were used in the studies, ranging from 398 [20] to 44,451 [19]. All the articles managed to statistically present the findings and clearly indicated whether the results were statistically significant or not using *p*-values and confidence intervals in other situations. 

In general, it was found that hand-washing interventions had resulted in a reduction in respiratory conditions, gastro-intestinal problems and school absenteeism in the intervention groups compared to the control groups. One of the studies [17], in which a comparison was made between waterless hand sanitizer and hand washing with water and soap, found that students at sanitizer schools were 23% less likely to have observed rhinorrhoea than control students (*p* = 0.02). By contrast, in another study [18], it was found that there was non-significant reduction in *E. coli* presence on the hands of children among intervention school pupils (soap for hand washing) compared with controls. Similarly, findings in yet another study revealed that a hygiene promotion and water treatment intervention did not reduce *E. coli* presence on the hands of children (RR (risk ratio) = 0.92, CI = 0.5–1.56) [13].

Seven studies found that hand washing had decreased the incidence of diarrhoea (53%–73%). Three studies found a decrease in acute respiratory infection (RR = 0.77; CI = 0.62–0.95; EDM (Exploratory Data Mining) −2%; 90% CI = −3% to −1%) but no significant decrease in the incidence of diarrhoea. A study by Pickering [17] found that hand snitizer was significantly better than hand washing with respect to reductions in levels of faecal streptococci (RR = 0.77; 95% CI = 0.62−0.95; *p*-value = 0.01). 

### 3.7. Factors Influencing Hand Washing

According to Dreibelbis (2014) [22], contextual, psychosocial and technological factors influence hand washing at five different aggregate levels: habitual, individual, interpersonal, community and societal [22]. There was interplay of all three factors in the implementation of interventions in all studies under this review to influence hand-washing behaviour, which were delivered at different levels. For example, under contextual factors, researchers made sure that they created a favourable environment for habit formation, with hand-washing stations put in place and soap made available [14,15]. Under psychosocial factors, researchers trained participants to trigger self-efficacy, increase knowledge, and induce a sense of perceived threat due to infections, and there was advocacy from the interventionist [13,16,17,18,20]. The technology factors, which were highly applicable to all the studies reviewed, were evident through financing, the availability of hand-washing facilities, accessibility, and demonstrations on hand-washing procedure and how to use hand-washing stations such as tippy-taps. This was demonstrated at all five levels, the habitual, individual, interpersonal, school community and structural levels. It was found that the studies reviewed used more technological factors than contextual and psychosocial factors.

### 3.8. Hand Hygiene Intervention Strategies

This systematic review identified and grouped the activities in the articles under review into three intervention strategies that were used in the implementation of hand-washing interventions. The identified intervention strategies that emerged are training/education, policy, and funding. 

### 3.9. Training

Training is a critical success factor and represents one of the cornerstones for improvement of hand hygiene practices [7]. Nearly all the eight studies under review had a training component. The only difference was the level at which it was implemented (multi-level). For example, different groups of people, such as teachers, nurses, parents, and field workers delivered the training to schoolchildren, and students were trained as trainers of trainers, together with their teachers. Children were told to wash their hands at recommended times, such as before and after eating, and after using the toilet. Schoolchildren require training on the importance of hand hygiene and the correct procedures for hand washing and hand rubbing. Clear education messages on hand washing help to induce behavioural and cultural change and ensure that competence is deep-rooted and maintained among all children in relation to hand-washing hygiene. While all studies had training components, no single study that used hand washing in its intervention described the hand-washing technique used in children’s training, that is whether they had used the conventional 7-step hand-washing technique base on the World Health Organization (WHO), or the simplified 5-step hand-washing technique, which has been tested and found to be effective in a study performed in Hong Kong [24,25]. Training is an important strategy that can be easily integrated with all other essential strategy components. [7]. 

### 3.10. Policy

Policy as an intervention strategy was visibly implemented in two ways, first by creating an institutional safety climate, and second by putting reminders in strategic places in the school environment. 

The institutional safety climate refers to creating an environment and perceptions that facilitate awareness-raising and consideration of hand-washing improvement as a high priority at all levels, including active participation at both the institutional and individual levels, as well as awareness of individual and institutional capacity to change and improve self-efficacy [7]. In most studies, empowerment was facilitated by training teachers, children and parents to be trainers of trainers. Schoolchildren were encouraged to make tippy-taps in a study performed in Uganda [20]. Also encouraged was the formation of health clubs, which were responsible for monitoring the use of hand hygiene resources. The involvement of schoolchildren encouraged active participation and ownership of the intervention strategy or program, thereby making it more likely to be sustained.

Reminders in the school setting are key tools for prompting and reminding children about the importance of hand washing, as well as about the appropriate indications and procedures for performing it [7]. All studies tried to deliver the content through a multimedia approach, which is encouraged when training children so that they are interested and thus motivated to get the most out of the lessons [24,25]. Posters were put in visible locations close to the toilet, eating and hand-washing places. Pamphlets were distributed to children and parents as reminders of when, why and how to wash hands.

### 3.11. Funding 

Funding ensures that schools have the necessary infrastructure in place to allow students to perform hand washing [7]. Compliance with hand washing among children is only possible if schools ensure that infrastructure and a reliable and permanent supply of hand hygiene products are available at the right times and in the right locations [7]. Almost all studies ensured that the experimental group was supplied with adequate resources, such as alcohol-based hand rubs and soap and water throughout, to make hand hygiene as easy and convenient as possible. For example, in one Pakistan study, workers supplied families with soap as needed [15]. All three projects conducted in Kenya had funding from an international organization to implement the Water Sanitation and Hygiene (WASH) project in their country, which helped to fund most of the resources. For example, they managed to install hand-washing facilities and to construct toilets [16,18]. Most studies used more than one strategy to implement hand hygiene, which made it difficult to isolate and conclude which type of strategy was most effective. However, the commonly used and most prioritized combination of strategies was training, followed by funding and policy.

### 3.12. Multi-Level Intervention

An intervention is multi-level if it addresses the individual client, as well as at least two levels of contextual influence, such as organizations and providers, thereby targeting at least three different sources of influence [10,11]. While multi-level interventions in health care are less robust, it is believed that they influence interdependent interaction, thereby producing desirable outcomes [10,11]. Therefore, this review attempted to look closely at the levels that were involved in the implementation of hand washing, and analysed them in order to determine whether multiple levels were used or not compared to the outcome of each study.

Although the studies did not clearly state that they used multi-level interventions, the review found that almost all had implemented their interventions at three different levels, thereby qualifying as multi-level interventions. Five studies out of eight had managed to implement a hand-washing intervention at four different levels by involving the individual participants, family and social support providers, and organizations. Two studies [14,15] were not very clear on how they involved the organizations/schools. However, they had managed to use three levels of intervention, as presented in Table 4 below. 

In general, six studies found that hand washing was associated with statistically significant reductions in diarrhoea, respiratory infection and school absenteeism. Incidence of diarrhoea decreased by 53%–73% in the reviewed articles. Respiratory infection decreased by risk ratio (RR) of 0.77; CI = 0.62–0.95; EDM −2%; 90% CI = −3% to −1%). Reductions in school absenteeism due to diarrhoea and influenza-like infection were 33% (*p* < 0.0001) and 40% (*p* < 0.0001) respectively. These findings are consistent with literature showing that using a multi-level intervention and a combination of strategies improves efficiency and effectiveness, and hence is likely to achieve the goals of the study/programme [10,11].

## 4. Discussion

This review identified multi-level hand hygiene intervention strategies used in the studies under review to reduce diarrhoea and respiratory infections among schoolchildren aged six to twelve in developing countries. Published research articles from online journal databases were analysed, finding a mixture of high-and low-quality RCT/CRCT studies. The methodological rigour was assessed using the Jadad Scale for reporting RCTs, with substantial flaws detected [21].

The results of the review revealed that hand washing can reduce the number of microorganisms on the hands, in turn reducing the incidence of diarrhoea, respiratory infection and school absenteeism [14,15,16,20]. This means that if correct hand-washing interventions can be scaled up globally, especially in developing countries, the lives of many children could be saved, and morbidity reduced. 

However, due to the limited number of quality studies done in developing countries, especially in Africa, doubt remains as to the generalizability of the effectiveness of hand-washing interventions. While this review acknowledges the few quality studies performed in developing countries, a lack of methodological rigour creates uncertainty about their findings. Methodological challenges were present, such as contamination of intervention and control groups [14,15], lack of blinding [13,16,17,18,19,20], lack of placebo use, and lack of a homogeneous setting [18]. In addition, the review found that none of the studies that used hand washing as an intervention had specified or described the hand-washing technique used, that is, whether they had used the conventional 7-step hand-washing technique base on the World Health Organization (WHO). None of the studies stated that they had used the simplified 5-step hand-washing technique, which has been tested and found to be effective in a study performed in Hong Kong [24,25].

Addressing challenges that affect hand washing among schoolchildren can be vital in promoting health and reducing school absenteeism due to infectious diseases. While studies in this review tried to address some of these factors in their implementation, contextual and psychosocial factors were seldom used. [22,23]. Future studies need to consider using all three types of factor and delivering interventions at all five levels to maximize their impact on behaviour change and increase the uptake of hand washing among schoolchildren. Of the three intervention strategies identified, training and funding were more commonly applied than policy. These strategies were not used in isolation in any of the studies, but rather in combination with other strategies; as such, the reviewed studies conformed and qualified as proponents of the multi-level intervention approach [7,10,11]. These findings can help to inform school health workers, enabling them to categorize and prioritize activities into viable strategies when implementing multi-modal hand-washing interventions.

Several limitations were present in this review. First, it is possible that other studies may have been under way but not published and not explored in the international trial registers, and that they were therefore left out of the review. Secondly, the reviewed studies were restricted to those written in English only.

## 5. Conclusions 

Apart from the identified strategies, the multi-level approach and factors that influence hand washing, this review adds to the bulk of existing evidence that hand-washing interventions can reduce the incidence of gastro-intestinal and respiratory conditions, as well as school absenteeism. However, more scientifically sound, evidence-based studies need to be carried out in developing countries; since few studies have been performed, it is difficult to generalize the findings. In addition, a simplified hand-washing technique, which has been shown to be effective in reducing school absenteeism in Hong Kong [24,25], needs to be carried out in developing countries to assess its efficacy, acceptability and sustainability. This may provide an alternative solution to conventional hand washing. Upcoming studies need to evaluate the effectiveness of using intervention strategies with a multi-level approach when addressing factors that affect hand washing to scale up and improve hand-washing practice among schoolchildren.

## Figures and Tables

**Table 1:** Diagnostics of included studies.

Author (Year) [Study ID]	Population				Intervention	Control	Study Design (Cluster RCT)	
	Participants	Age	Setting	Country			Cluster	Number of Clusters
Greene et al. (2012) [13]	School children	6–16 years	Public primary school	Nyanza Province in Kenya	Hygiene promotion & water treatment (12 schools) Hygiene promotion & water treatment PLUS 7 new Ventilated Improved Pits (5 schools)	No intervention BUT to receive at conclusion of study	Public primary school	17 control 17 intervention
Luby et al. (2005) [14]	Children	<15 years	Squatter settlement	Pakistan	Hand washing Antibacterial soap Plain soap	Not clear	Neighbourhood and household	25 intervention, 11 control neighbourhoods & 600 intervention; 300 control households
Luby et al. (2004) [15]	Children	<15 years	Urban squatter	Karachi Pakistan	Hand washing Antibacterial soap Plain soap	Standard practice Children’s books, pens, pencils	Neighbourhoods *n =* 72	300 household intervention 1 300 household intervention 2 306 household control
Patel et al. (2012) [16]	Children	School age & 6–35 months old in villages	Primary schools (*n* = 43);Villages (*n =* 60)	Kenya (Rural)	Hand washing Installation of water stations near latrine for hand washing and classroom for drinking	NICHE Project	School and villages	30 intervention & 30 control villages; 21 intervention & 22 control schools; 312 intervention households & 331 comparison households
Pickering et al. (2013) [17]	Primary school children (*n =* 1364)	2–13 years	Schools in urban settings (*n* = 6)	Kenya	Hand washing with soap Alcohol-based hand sanitizer	No intervention	Schools	6 schools; 2 in each of the three arms
Saboori et al. (2013) [18]	Pupils	Primary school age	Public primary school (*n* = 60)	Kisumu/Nyando and Rachuonyo Districts in Kenya	Hand washing Latrine cleaning plus hand washing	No intervention	Public primary schools	577 pupils’ intervention 1 570 pupils’ intervention 2 578 pupils’ control 20 schools in each arm
Talaat et al. (2011) [19]	Elementary school children	Primary school age Median age 8 years	Government schools (*n* = 60)	Cairo in Egypt	Intensive hand hygiene campaign	Not clear	Schools	30 intervention 30 control
Zgang et al. (2013) [20]	Elementary school children	Elementary school age	Elementary school	Uganda	Tippy-taps Soap Complementary education	Initially only received education program	Primary schools	200 intervention control

**Table 2:** Search strategy.

Databases	Search	Search Words	Number of Retrieved Studies	Number of Qualified Studies
CINAHL	Title & abstract	Effect*; Hand hygiene OR Hand wash OR Hand disinfection; Intervention OR Strategy OR Technique; AND Schoolchildren.	524	0 qualified
Web of Science	Title & abstract	Effect*; Hand hygiene OR Hand wash OR Hand disinfection; Intervention OR Strategy OR Technique; AND Schoolchildren.	8676	0 qualified
PsychInfo (via ProQuest)	Title, abstract & full article	Effect*; Hand hygiene OR Hand wash OR Hand disinfection; Intervention OR Strategy OR Technique; AND Schoolchildren.	2922	1 qualified
MEDLINE (via EBSCOhost)	Title, abstract & full article	Effect*; Hand hygiene OR Hand wash OR Hand disinfection; Intervention OR Strategy OR Technique; AND Schoolchildren.	1429	7 qualified
Pub Med	Title, abstract & full article	PubMed; search terms (Mesh) (“hand hygiene” (MeSH Terms) OR (“hand” (All Fields) AND “hygiene” (All Fields)) OR “hand hygiene”(All Fields)) OR (“hand disinfection” (MeSH Terms) OR (“hand” (All Fields) AND “disinfection” (All Fields)) OR “hand disinfection” (All Fields) OR (“hand” (All Fields) AND “washing” (All Fields)) OR “hand washing” (All Fields)) OR ((“hand” (MeSH Terms) OR “hand” (All Fields)) AND (“disinfectants” (Pharmacological Action) OR “disinfectants” (MeSH Terms) OR “disinfectants” (All Fields) OR “disinfectant” (All Fields))) AND “humans” (MeSH Terms)	7552	1 qualified
Total number of unique studies Total number of qualified studies				9 articles 8 articles

**Table 3:** JADAD scores for the included studies.

Authors	Year	Reference	Randomization	Blinding	Account of all Participants	Total = 5
1. Greene et al.	2012	[13]	1	0	1	2
2. Luby et al.	2005	[14]	2	1	1	4
3. Luby et al.	2004	[15]	2	1	1	4
4. Patel et al.	2012	[16]	1	0	1	2
5. Pickering et al	2013	[17]	1	0	1	2
6. Saboori et al.	2013	[18]	1	0	0	1
7. Talaat et al.	2011	[19]	2	0	0	2
8. Zhang et al.	2012	[20]	2	0	0	2

**Table 4:** Multi-level interventions and strategies used in the included studies.

Author (Year) [Study ID]	Multi-Level				Strategies			Outcome of Study
	Individual	Family & Social Support	ProviDer/Team	Organization/Practice Setting	Training/Education	Funding/System Change	Policy (Reminder/Climate)	
Greene et al. (2012) [13]	√	√	√	√	√	√	√	Hygiene promotion had no impact on presence of any *E. coli* hand contamination (RR = 1.1; 95% CI = 0.7–1.8)
Luby et al. (2004) [15]	√	√	√	X	√	√	√	Lower incidence of diarrhoea was 57% (95% CI = –73% to –41%)
Luby et al. (2005) [14]	√	√	√	X	√	√	√	Lower incidence of diarrhoea was 53% (CI = –65% to –34%) and impetigo was 34% (CI = –52% to–16%)
Patel et al. (2012) [16]	√	√	√	√	√	√	X	Decrease in ARI (EDM –2%; 90% CI = –3% to –1%) but not in diarrhoea (EDM 0%; 90% CI = 0% to 0%)
Pickering et al. (2013) [17]	√	√	√	√	√	√	√	Hand sanitizer better than hand washing in reducing rhinorrhoea (RR = 0.77; CI = 0.62–0.95). Any loose stool (RR = 0.80; CI = 0.67–0.95). Soap better than sanitizer (RR = 0.77; CI = 0.62–0.95).
Saboori et al. (2013) [18]	√	√	√	√	√	√	√	Hand washing had non-significant effect on reduction of *E. coli* contamination (OR = 0.43; CI = 0.15–1.23)
Talaat et al. (2011) [19]	√	√	√	√	√	√	X	Reductions in school absenteeism due to diarrhoea was 33% (*p* < 0.0001) and influenza-like infection was 40% (*p* < 0.0001)
Zhang et al. (2012) [20]	√	√	√	√	√	√	X	Absence of stomach pain (proxy measure for diarrhoea) (*t* = 10.8; 95% CI = 0.92–1.68)

Key: √ = Intervention or strategy was used in the study; X = Intervention or strategy was not used in the study; RR: relative risk; OR: odds ratio.
